# Correction of Nasolabial Folds Using Polydioxanone Cog Threads Combined With Botulinum Toxin Type A: A Prospective Comparative Clinical Study

**DOI:** 10.7759/cureus.101158

**Published:** 2026-01-09

**Authors:** Swapnil U Shinde, Samir Mansuri, Neelam Choudhary, Prashant Pillai, Arun Deepak, Rahul VC Tiwari, Heena Dixit, Seema Gupta

**Affiliations:** 1 Department of Oral and Maxillofacial Surgery, Bharati Vidyapeeth (Deemed to be University) Dental College and Hospital, Sangli, IND; 2 Department of Oral and Maxillofacial Surgery, Al Kuwait Hospital, Dubai, ARE; 3 Department of Oral and Maxillofacial Surgery, RKDF Dental College and Research Centre, Bhopal, IND; 4 Department of Orthodontics, RVS Dental College and Hospital, Coimbatore, IND; 5 Department of Blood Cell, Commissionerate of Health and Family Welfare, Hyderabad, IND; 6 Department of Orthodontics, Kothiwal Dental College and Research Centre, Moradabad, IND

**Keywords:** botulinum toxin, cosmetic, face, nasolabial, threads

## Abstract

Introduction

Nasolabial folds (NLFs) remain one of the most common concerns that drive patients toward minimally invasive facial rejuvenation. This prospective observational study aimed to compare the clinical efficacy and durability of polydioxanone (PDO) cog thread lifting alone and in combination with botulinum toxin type A (BoNT-A) for the correction of moderate NLFs.

Materials and methods

Thirty-two patients aged 40-65 years with Modified Fitzpatrick Wrinkle Scale (MFWS) grades 2-3 nasolabial folds were divided into two groups. Group A (n = 16) received PDO cog thread lifting alone, using bidirectional barbed threads inserted along the midfacial lifting vectors. Group B (n = 16) underwent an identical thread-lift procedure, followed by targeted BoNT-A injections into the perioral and nasolabial musculature 1h later. Standardized outcomes were assessed at baseline, 15 days, 3 months, and 6 months using wrinkle depth measurement, MFWS, the Global Aesthetic Improvement Scale (GAIS), patient satisfaction score, and complication profiling.

Results

Both groups showed early improvement; however, the combination group (Group B) achieved significantly greater reductions in wrinkle depth and MFWS scores, along with higher GAIS and patient satisfaction ratings at the 3- and 6-month follow-ups. Two-way analysis of variance (ANOVA) confirmed a significant group × time interaction favoring sustained superiority in the combined treatment arm. The complication profiles were comparable and consisted only of mild, self-limiting bruising, swelling, and temporary dimpling, with no serious adverse events.

Conclusion

Adjunctive BoNT-A significantly enhanced and prolonged the esthetic outcomes of PDO cog thread lifting for nasolabial folds by mitigating the dynamic muscular forces that counteract mechanical elevation. The combined protocol is safe, effective, and offers a synergistic minimally invasive strategy that provides more natural and durable midfacial rejuvenation than thread-lifting monotherapy.

## Introduction

Facial aging is a progressive, multifactorial process characterized by structural changes in the skin, soft tissues, retaining ligaments, and underlying skeletal support. Patients commonly notice early manifestations such as dryness, thinning of the dermis, and dynamic lines that gradually evolve into deeper and more permanent wrinkles [1]. Among these, the nasolabial fold (NLF) is one of the most prominent age-related features, and a major reason individuals seek aesthetic correction. The deepening of the NLF occurs due to descent of the malar fat pad, attenuation of midfacial retaining ligaments, repetitive muscular activity, and age-related remodeling of the midfacial skeleton [2]. Together, these alterations produce a heavier midface and sharper fold contour, prompting patients to pursue minimally invasive facial rejuvenation.

Polydioxanone (PDO) cog thread lifting has emerged as an effective and minimally invasive technique for repositioning sagging tissues by engaging the superficial musculoaponeurotic system (SMAS) [3]. This method provides an immediate mechanical lifting effect followed by neocollagenesis, which improves midfacial support over subsequent months. Botulinum Toxin A (BoNT-A) has been widely used to reduce hyperdynamic muscular contraction by inhibiting acetylcholine release at the neuromuscular junction [4]. Relaxing muscle activity in the perioral and nasolabial regions may enhance the longevity of thread-lift outcomes by minimizing downward muscular pull.

While both techniques have independently demonstrated efficacy, there is limited evidence evaluating whether combining PDO thread lifting with BoNT-A offers superior correction of NLF compared to thread lifting alone [5,6]. Although PDO cog thread lifting effectively corrects the static component of nasolabial folds by repositioning sagging tissues, it does not address the dynamic muscular forces that contribute to fold recurrence. BoNT-A temporarily reduces perioral muscle activity, thereby minimizing antagonistic pull on the lifted tissues during the healing and collagen remodeling phase. When used adjunctively, BoNT-A serves to stabilize and prolong the aesthetic outcome of thread lifting rather than act as a redundant or standalone treatment. Considering that NLF is influenced by both gravitational descent and dynamic muscle activity, a multimodal approach could theoretically provide more stable and refined results.

The aim of the present study was to comparatively evaluate the clinical effectiveness of PDO cog thread lifting alone and in combination with BoNT-A for NLF correction. Specifically, this study sought to assess improvements in wrinkle depth, Modified Fitzpatrick Wrinkle Scale (MFWS) scores, Global Aesthetic Improvement Scale (GAIS) ratings, and patient satisfaction across both treatment groups, while also determining whether the adjunctive use of BoNT-A enhances the stability and overall esthetic outcome of the thread lift. Additionally, this study aimed to monitor and document any procedure-related complications to provide a comprehensive understanding of the safety and therapeutic advantages of this dual-modality approach.

## Materials and methods

Study design and setting

This study was conducted as a prospective observational clinical study in which the investigator recorded standardized readings on patients undergoing nasolabial fold correction procedures in the Department of Oral and Maxillofacial Surgery, RKDF Dental College and Research Centre, Bhopal, India. This study was conducted over a period of 12 months (Aug 2024 - Aug 2025). No part of the clinical procedure was modified for research purposes; the investigator only observed and documented the pre- and post-treatment findings. Ethical approval was obtained from the Institutional Ethics Committee (RKDF/DC/PG/2024/143), and written informed consent was obtained from all participants before enrolment, including consent for clinical photography and scoring.

Eligibility criteria

Patients aged 40-65 years with visible NLFs graded between 2 and 3 on the MFWS [7] were considered eligible. Only medically stable individuals willing to undergo the procedure and attend all scheduled follow-up visits were included. Patients with unrealistic esthetic expectations; local skin infections; known hypersensitivity to lidocaine, epinephrine, PDO material, or BoNT-A; and those medically unfit for minor esthetic interventions were excluded. Individuals outside the required age group or those with NLF severity outside the MFWS 2-3 range were not included.

The required sample size was calculated using the G*Power software (version 3.1.9.2; Heinrich Heine University, Düsseldorf, Germany). The sample size was determined by using wrinkle depth reduction as the primary outcome measure. Based on an expected mean difference of 0.4 mm, a standard deviation of 0.35 mm [7], an alpha of 0.05, and 80% statistical power, approximately 12 patients were required per group. Accounting for an anticipated 20% dropout rate, the final target enrolment was set at 16 participants per group, or 32 subjects for the study. Eligible patients were consecutively enrolled during the study period and assigned to one of the two treatment groups based on the treatment protocol selected after routine clinical consultation. Patients who consented to undergo PDO cog thread lifting alone were allocated to Group A, while those who consented to receive PDO cog thread lifting followed by adjunctive botulinum toxin type A injections were allocated to Group B. Each group comprised 16 patients.

Clinical assessment tools

The baseline evaluation included measurement of wrinkle depth, MFWS score [8], and GAIS grading [9]. MFWS is a validated wrinkle-severity grading tool that classifies NLFs into five ordered categories based on depth, visibility, and contour changes. Scores range from Grade 0 (no wrinkles) to Grade 4 (deep, severe wrinkles with pronounced fold shadows), allowing consistent and reproducible assessment of wrinkle severity across clinical settings. The scale is simple, clinically intuitive, and free to use for research, without licensing or permission requirements.

Similarly, GAIS is a widely used physician- and patient-reported outcome measure that evaluates overall aesthetic enhancement after treatment. The GAIS uses a 5-point ordinal scoring system ranging from “Very Much Improved” to “Worse,” reflecting the degree of visible improvement relative to the baseline. A score of “Very Much Improved” indicates optimal cosmetic change, whereas “No Change” or “Worse” reflects minimal or negative aesthetic response. GAIS is also free for academic use and requires no special permission.

Pre-procedure preparation

Each patient underwent standardized skin preparation, which included cleansing with a 2% chlorhexidine solution (Hibiscrub®, Mölnlycke Health Care, Gothenburg, Sweden). Topical anesthesia was applied (EMLA® cream, AstraZeneca PLC, Cambridge, UK) for approximately 45-60 min. After cream removal, local anesthesia was administered using 2% lidocaine with epinephrine 1:200,000 (Aspen Pharmacare Holdings Ltd., Durban, South Africa). Prior to the procedure, skin markings were performed with the patient in an upright position to counteract gravitational tissue displacement. Standardized lifting vectors were drawn from the temporal entry point toward the nasolabial fold, following the direction of midfacial soft-tissue descent. These markings guided thread trajectory, ensured symmetry, and helped achieve consistent and reproducible tissue elevation across patients.

Procedure in Group A: PDO cog thread lift alone

In Group A, correction of the NLF was performed exclusively using PDO barbed threads (MINT™ PDO threads; HansBiomed Co. Ltd., Seoul, South Korea). After adequate anesthesia, a small stab incision was made in the temporal hollow, approximately 1 cm lateral to the lateral orbital rim, using an 18-gauge needle. Through this entry site, two precannulated PDO cog threads were introduced into the subcutaneous plane along pre-marked lifting vectors. The cannula was advanced carefully until the barbs were engaged in the deep medial fat pad and SMAS. Once optimal placement was confirmed, gentle superior traction was applied to elevate the sagging midfacial tissue and reduce the depth of the nasolabial fold. The cannula was then withdrawn, excess thread was trimmed, and the entry point was manually compressed to minimize dimpling. The procedure was repeated on the contralateral side using the same technique (Figure 1).

**Figure 1:**
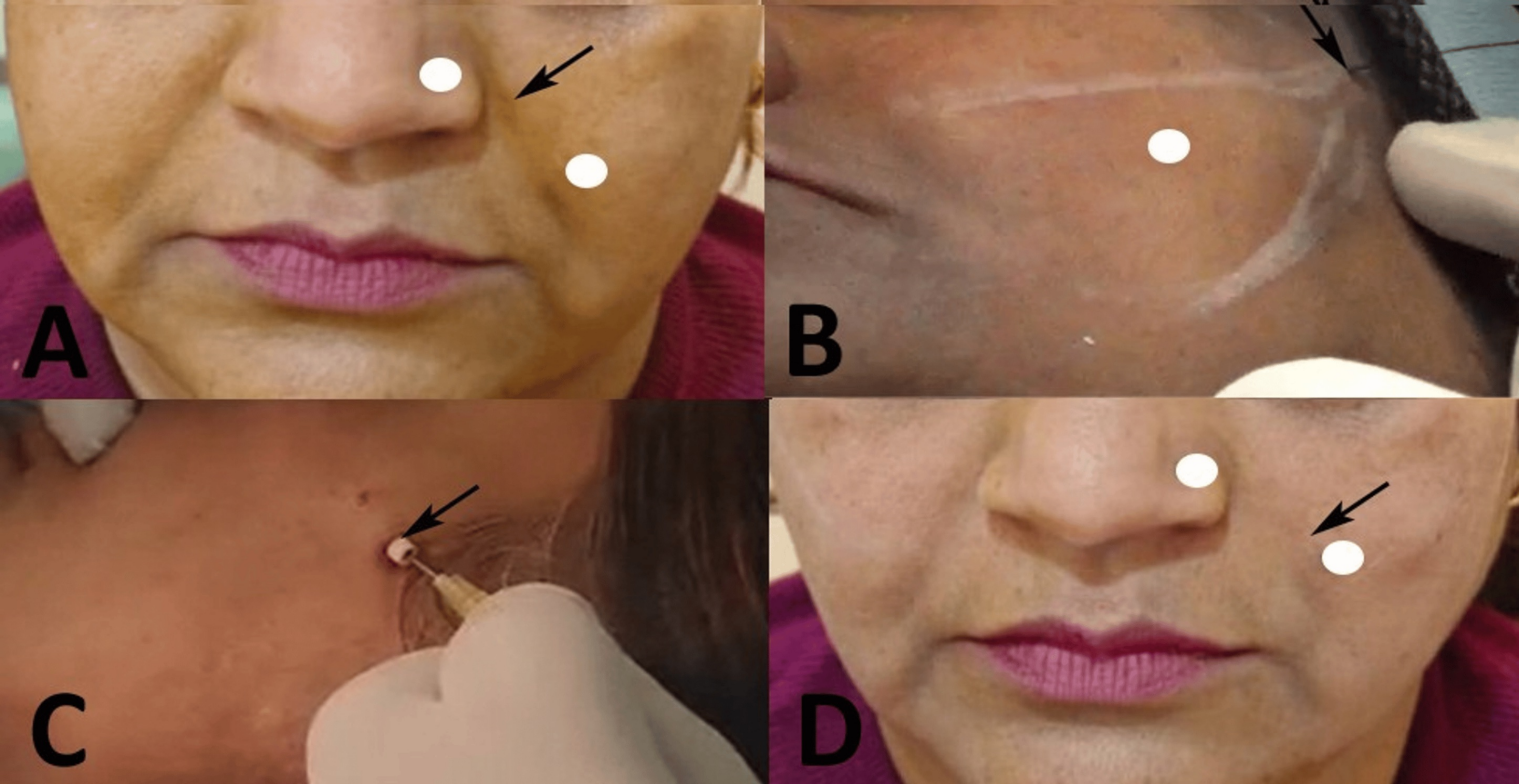
Representative patient from Group A: PDO cog thread lifting monotherapy (A) Baseline frontal view showing bilateral moderate nasolabial folds (MFWS Grade 3), (B) Intraoperative view showing temporal entry point for insertion of bidirectional barbed PDO cog threads, (C) Immediate post-procedure photograph during administration of supplemental local anesthetic (2% lidocaine with 1:200,000 epinephrine) along the thread tracts for enhanced post-operative comfort and hemostasis, (D) 6-month follow-up frontal view demonstrating good initial correction with partial recurrence of the nasolabial folds. Original images of the patient. Used with the patient's permission. PDO: polydioxanone; MFWS: Modified Fitzpatrick Wrinkle Scale

Procedure in Group B: PDO cog thread lift combined with BoNT-A

Group B patients underwent the same standardized thread-lift procedure as described for Group A, using identical thread types, insertion vectors, and lifting maneuvers. Approximately one hour after thread placement, BoNT-A injections were administered (BOTOX®; Allergan Aesthetics, AbbVie Inc., North Chicago, IL, USA). The toxin was reconstituted with 10 mL of preservative-free normal saline and 3 mL of lidocaine with epinephrine, providing a total volume of 13 mL. Injections were delivered at a dose of approximately 1.5 U per cm of thread length, placed 3-4 mm lateral to the thread vector to target the underlying muscles that contribute to NLF formation. Additional focused injections were delivered into the levator labii superioris alaeque nasi muscle using a palpation-guided approach to reduce the vertical pull on the fold. A 31-gauge insulin syringe (BD, Franklin Lakes, New Jersey, USA) was used for precise dosing and to minimize discomfort (Figure 2).

**Figure 2:**
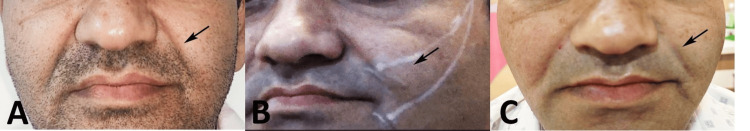
Representative patient from Group B: PDO cog thread lifting combined with botulinum toxin type A (A) Baseline frontal view showing bilateral moderate nasolabial folds (MFWS Grade 3), (B) Intraoperative view showing temporal entry point for insertion of PDO cog threads and botulinum toxin type A injections, (C) 6-month follow-up frontal view demonstrating superior and sustained softening of the nasolabial folds with excellent long-term esthetic outcome. Original images of the patient. Used with the patient's permission. PDO: polydioxanone; MFWS: Modified Fitzpatrick Wrinkle Scale

Follow-up and outcome recording

All clinical outcomes were recorded at 15 days, 3 months, and 6 months, using standardized photography and consistent scoring protocols. Wrinkle depth was assessed through digital measurements, MFWS scores were assigned by clinical grading, and GAIS scores were documented according to recognized descriptors. Any complications, such as bruising, swelling, dimpling, asymmetry, or thread extrusion, were documented systematically and managed according to standard clinical protocols. Post-procedural complications were monitored at all scheduled follow-up visits (15 days, 3 months, and 6 months). For analytical purposes, complications were analyzed cumulatively over the entire 6-month follow-up period. Each complication was recorded once per patient, irrespective of the time point at which it first appeared. Patients experiencing the same complication at multiple follow-up visits were counted only once for that specific complication. This approach was adopted to reflect the overall safety profile of the interventions rather than time-specific event frequency.

Patient satisfaction was assessed using a 5-point Patient Satisfaction Scale (PSS) [10], a simple Likert-type tool widely used in aesthetic outcome research to quantify a patient’s subjective improvement after treatment. The scale ranges from 1 (very dissatisfied) to 5 (very satisfied), providing a direct measure of postprocedural satisfaction. PSS is freely available for academic use and does not require licensing or special permission for incorporation into clinical research.

Statistical analysis

Statistical analysis was conducted using the Statistical Package for Social Sciences (SPSS) version 23.0 (IBM Corp., Armonk, New York, USA). Continuous and ordinal data that followed a normal distribution, as confirmed by the Shapiro-Wilk test, were summarized as the mean ± standard deviation. Categorical complication data were presented as frequencies and percentages. Intergroup comparisons at each time point were performed using independent-sample t-tests. Two-way analysis of variance (ANOVA) was employed to evaluate the effects of group and time simultaneously. Statistical significance was defined as p < 0.05, and p < 0.01 indicated highly significant results.

## Results

The demographic variables of the two groups were comparable at baseline. There was no statistically significant difference in age between the groups (Group 1: 35.02 ± 5.75 years vs. Group 2: 32.56 ± 7.34 years). The sex distribution was also similar, with no significant difference in the proportion of males or females between the groups (p = 0.285), as given in Table 1.

**Table 1:** Demographic details of the study groups. N (%): number of patients presented as frequency and percentage; age was compared with the independent t-test, while the chi-square test was used to compare sex distribution. p > 0.05 denotes no statistical difference.

Parameters		Group 1	Group 2	Test statistics	p-value
Age	Years (Mean ± SD)	35.02 ± 5.75	32.56 ± 7.34	1.05	0.299
Sex	Male N (%)	6 (37.50)	7 (43.75)	1.12	0.285
	Female N (%)	10 (62.50)	9 (56.25)		

The results demonstrated that both treatment groups showed improvement in NLF appearance; however, Group B achieved superior and more sustained outcomes. At all post-treatment intervals (15, 90, and 180 days), Group B exhibited lower wrinkle depth, better MFWS scores, higher clinician-rated esthetic improvement (GAIS), and greater patient satisfaction (PSS) than Group A. The most notable differences were observed at the 90- and 180-day marks, suggesting that the combined protocol provided longer-lasting effects (Table 2).

**Table 2:** Descriptive analysis of outcome parameters at various time points. Values are presented as mean ± standard deviation (SD). N: number of patients in group; MFWS: Modified Fitzpatrick Wrinkle Scale scores (0-4) [8]; GAIS: Global Aesthetic Improvement Scale (0-4) [9]; PSS: Patient Satisfaction Scale (1-5) [10]; Group A: Nasolabial fold treated with polydioxanone (PDO) cog thread thread; Group B: Nasolabial fold treated with Botulinum Toxin A (BoNT-A) and PDO cog thread lift; NA: the measure is not applicable at baseline or not assessed at that time point.

Parameter	Group	N	Baseline	15 days	90 days	180 days
Wrinkle depth (mm)	A	16	2.0 ± 0.3	1.2 ± 0.2	1.5 ± 0.3	1.8 ± 0.3
	B	16	2.0 ± 0.3	1.0 ± 0.2	1.1 ± 0.2	1.5 ± 0.3
MFWS (0–4)	A	16	2.5 ± 0.5	1.5 ± 0.5	1.8 ± 0.4	2.2 ± 0.5
	B	16	2.5 ± 0.5	1.2 ± 0.4	1.3 ± 0.4	1.8 ± 0.5
GAIS (0–4)	A	16	0	2.0 ± 0.6	1.5 ± 0.5	1.0 ± 0.4
	B	16	0	2.5 ± 0.5	2.2 ± 0.5	1.5 ± 0.5
PSS (1–5)	A	16	NA	4.2 ± 0.6	3.8 ± 0.7	3.0 ± 0.8
	B	16		4.5 ± 0.5	4.2 ± 0.6	3.5 ± 0.7

The results demonstrated a significant Group × Time interaction effect (Two-way ANOVA, p<0.001) for all outcomes except PSS, indicating that the treatment trajectories differed meaningfully between the groups. Independent t-tests revealed that, compared to Group A, Group B achieved significantly greater improvement in wrinkle depth at 15, 90, and 180 days (all p < 0.01). Similarly, patient and physician assessments (MFWS, GAIS) showed significantly better outcomes in Group B from 90 days onward (p < 0.05), with GAIS showing earlier significance at 15 days. However, no significant inter-group difference was found for PSS at any time point. The inference is that the intervention in Group B produced a clinically superior and sustained reduction in wrinkle depth and associated aesthetic scales compared to Group A, although this objective and observer-rated superiority did not translate into a statistically significant difference in the patients' own subjective satisfaction over the 180-day period (Table 3).

**Table 3:** Comparison of outcome parameters between groups at different time points using independent samples t-test and two-way analysis of variance (ANOVA). *p < 0.05 considered statistically significant, p < 0.01 indicates highly significant results; **Two-way ANOVA used to assess overall Group × Time interaction effect. The independent t-test was used for between-group comparison at each time point. MFWS: Modified Fitzpatrick Wrinkle Scale scores [8]; GAIS: Global Aesthetic Improvement Scale [9]; PSS: Patient Satisfaction Scale [10]; NA: parameters not measured at baseline (GAIS, PSS).

Measure	Baseline		15 days		90 days		180 days		Overall Group × Time**	
	t-value	p-value	t-value	p-value	t-value	p-value	t value	p-value	F-value	p-value
Wrinkle depth	0.34	0.989	2.78	0.008*	4.37	0.001*	2.67	0.008*	21.45	0.001*
MFWS	0.68	0.992	1.83	0.070*	3.53	0.001*	2.26	0.031*	18.78	0.001*
GAIS	NA	NA	2.56	0.015*	3.95	0.004*	3.12	0.003*	15.92	0.015*
PSS	NA	NA	1.53	0.134	1.73	0.092	1.88	0.069	2.57	0.061

Complication rates, including bruising, swelling, and dimpling, were slightly higher in Group B; however, they were generally transient and manageable. These findings indicate that adjunctive BoNT-A enhanced and prolonged the efficacy of thread lifting for nasolabial folds, with a favorable risk-benefit profile. The analysis of treatment-related complications revealed comparable safety profiles between the groups. Although Group B exhibited numerically higher rates of bruising and swelling, these differences were not statistically significant (p = 0.445). Similarly, the incidences of dimpling, asymmetry, and thread extrusion showed no significant intergroup variation. All chi-squared statistics were low, with p-values exceeding 0.05, confirming that none of the observed differences in complication frequencies reached statistical significance (Table 4).

**Table 4:** Complications analyzed between groups using the chi-square test. Values presented as n (%) for each complication, χ² statistics compare the proportion of complications between groups. Data represent cumulative complications recorded across all follow-up visits (15 days, 3 months, and 6 months). Each patient was counted only once per complication, regardless of the number of follow-up visits at which the event was observed. p > 0.05 denotes no statistical significance. Group A: Nasolabial fold treated with polydioxanone (PDO) cog thread lift; Group B: Nasolabial fold treated with Botulinum Toxin A (BoNT-A) and polydioxanone (PDO) cog thread lift.

Complications	Group A		Group B		Chi-square stats	p-value
	Yes	No	Yes	No		
Bruising	4 (25%)	12 (75%)	6 (37.5%)	10 (62.5%)	0.582	0.445
Swelling	4 (25%)	12 (75%)	6 (37.5%)	10 (62.5%)	0.582	0.445
Dimpling	3 (18.7%)	13 (81.3)	3 (18.7%)	13 (81.3)	0	1
Asymmetry	3 (18.7%)	13 (81.3)	4 (25%)	12 (75%)	0.183	0.669
Thread extrusion	2 (12.5%)	14 (87.5%)	2 (12.5%)	14 (87.5%)	0	1

## Discussion

The findings of this prospective observational study highlight the enhanced clinical efficacy of combining PDO cog thread lifting with BoNT-A for NLF correction compared to PDO thread lifting alone. Both treatment groups demonstrated improvements in NLF appearance, but Group B (combination therapy) exhibited significantly greater reductions in wrinkle depth, lower MFWS scores, higher GAIS ratings, and superior patient satisfaction as measured by the PSS at 3 and 6 months post-treatment. These differences were most pronounced at later follow-up points, suggesting that adjunctive BoNT-A contributes to the prolonged stability of esthetic outcomes. Two-way ANOVA further confirmed the findings, underscoring divergent treatment trajectories favoring the multimodal approach.

These results can be attributed to the complementary mechanisms of these two modalities. PDO threads provide immediate mechanical elevation of sagging midfacial tissues by engaging the SMAS and malar fat pad, followed by neocollagenesis, which enhances tissue support over time [3]. However, gravitational descent alone does not fully account for NLF deepening; repetitive contractions of muscles, such as the levator labii superioris alaeque nasi and zygomaticus, contribute to dynamic wrinkling and downward pull, potentially undermining thread longevity [2,11]. BoNT-A inhibits acetylcholine release at the neuromuscular junction, thereby relaxing hyperdynamic musculature and reducing tension on lifted tissues [4]. This synergy likely explains the sustained improvements observed in Group B, as BoNT-A minimizes forces that could otherwise accelerate the fold recurrence.

Our observations align with prior research, emphasizing the benefits of combining thread lifting with neuromodulators for facial rejuvenation. For example, Ziade et al. [12] evaluated a triple combination of barbed threads, hyaluronic acid fillers, and BoNT-A for nonsurgical rhinoplasty in 30 patients and reported superior facial assessment and cosmetic enhancement quality of life questionnaire (FACE-Q) satisfaction scores and minimal efficacy decay over 1 year in the full combination group compared with threads plus BoNT-A alone. Although their focus was on nasal reshaping, the enhanced longevity from BoNT-A muscle relaxation mirrors our findings for NLFs, where dynamic components play an analogous role.

Similarly, Wanitphakdeedecha et al. [4] demonstrated that intradermal BoNT-A injections alone achieved significant facial lifting in 17 patients (77.3%). Their results support BoNT-A's standalone efficacy against dynamic aging; however, our study extends this by showing additive benefits when paired with threads, yielding more comprehensive midface repositioning. Similar to our findings, Binder [13] demonstrated the long-term effects of BoNT-A for facial lines, with no adverse effects even if given regularly for 13 years.

In contrast, studies on monotherapy provided a context for our comparative design. Germani et al. [14] conducted a randomized trial of PDO threads for facial lifting in 22 patients and found no significant difference in outcomes between six and 12 threads per face, with initial volumetric improvements diminishing by 60 days. This echoes the trajectory in Group A, where early gains in wrinkle depth and MFWS waned by six months, highlighting the limitations of threads alone in countering ongoing muscular activity.

The preliminary cranial tissue displacement observed over the initial period, commonly referred to as the lifting effect, is contingent on the primary tension engendered by the traction provided by the PDO threads. Thereafter, a caudal counter-displacement force, influenced by gravitational pull, tissue weight, and facial expressions, became increasingly evident. This caudal counter-displacement phenomenon has the potential to counteract the initial lifting effect, resulting in an outcome that is contrary to the intended effect because PDO threads lack anchorage to a rigid structural entity [3,15].

Regarding safety, complication rates were comparable between the groups, with transient bruising, swelling, and dimpling predominating but resolving without intervention. Group B showed numerically higher incidences, likely due to additional injections, but the differences were not statistically significant, which is consistent with the literature on minimally invasive procedures. Ziade et al. [12] reported higher adverse effect scores in thread-involved groups, attributing them to invasiveness, yet all resolved within a week. Similarly, Germani et al. [14] noted only minor self-limiting pain. These parallels confirm the favorable risk-benefit profile of our combination protocol, with no severe events such as thread extrusion or infection observed.

The Global Aesthetics Consensus Group proposed that the mid and lower regions of the facial structure exhibit the greatest responsiveness to a synergistic intervention involving both dermal fillers and botulinum toxin. It has been observed that a synergistic effect can be attained through meticulously orchestrated individualized treatment protocols. This methodology is not exclusively pertinent to the lower facial area; however, it is also applicable to the upper facial regions, as well as certain segments of the midface. The advocated strategy is personalization, meticulously designed to address the specific requirements of the patient [16].

Clinical implications

The superior outcomes of PDO threads plus BoNT-A suggest that this approach is the preferred first-line option for patients aged 40-65 years with moderate NLFs, particularly for those seeking minimally invasive rejuvenation with enhanced longevity. By integrating mechanical lift with muscle relaxation, clinicians can achieve more natural and stable results, potentially reducing the need for frequent touchups and improving cost-effectiveness. This is especially relevant in oral and maxillofacial surgery settings, where precise knowledge of midfacial anatomy can optimize vector planning and injection sites. Patient counseling should emphasize the additive benefits of screening for BoNT-A hypersensitivity to ensure safety.

Limitations

This study had several limitations. The observational design lacked randomization and introduced potential selection bias despite baseline similarity. The sample size was modest, powered for wrinkle depth, but possibly underpowered for rare complications. As this was a single-center study in India, generalizability to diverse populations may be limited by ethnic variations in facial aging. Follow-up was restricted to 6 months; longer-term assessment (12-24 months) is needed to evaluate durability during ongoing collagen remodeling. Finally, subjective scales are prone to assessor and patient bias, although standardized photography mitigates this bias.

## Conclusions

This study demonstrated that combining PDO cog thread lifting with BoNT-A yielded superior, longer-lasting correction of NLFs than thread lifting alone. The adjunctive use of BoNT-A effectively reduced the dynamic muscular pull and enhanced the stability and esthetic outcome of the mechanical lift. Both approaches proved safe with only mild transient complications. The synergistic dual-modality protocol addresses the multifactorial nature of nasolabial fold deepening more comprehensively than monotherapy does. It represents a reliable, minimally invasive option offering enhanced durability and natural results, making it a valuable addition to contemporary esthetic practices for midface rejuvenation.
